# Dexamethasone and compliance affect TTFields efficacy to glioblastoma patients: a systematic review and meta-analysis

**DOI:** 10.1186/s41016-022-00294-0

**Published:** 2022-09-03

**Authors:** Shupeng Li, Jiawei Dong, Xinyu Wang, Xiangqi Meng, Chuanlu Jiang, Jinquan Cai

**Affiliations:** Department of Neurosurgery, The Second Affiliated Hospital of Harbin Medical University, Harbin, 150086 China

**Keywords:** TTFields, Glioblastoma, Dexamethasone, Compliance, Meta-analysis

## Abstract

TTFields is a novel treating modality of glioblastoma (GBM) which can significantly prolong the overall survival (OS) of newly diagnosed or recurrent glioblastoma. Some researchers have revealed that a variety of factors can affect the efficacy of TTFields. So, we review the available literature about the influencing factors on efficacy of TTFields and then choose two experimentally supported factors: the dose of dexamethasone and compliance of TTFields to perform a meta-analysis. The PubMed, Embase, and the Cochrane Library are searched. Five articles are identified between 2014 and 2017. Three articles are about the compliance of TTFields. Two articles are about the dose of dexamethasone. The Newcastle-Ottawa Quality Assessment Scale (NOS) is used as an assessment tool to evaluate the methodological quality of all included trials. The scale’s range varies from 0 to 9 stars. According to the Cochrane Handbook for Systematic Reviews of Interventions, articles are graded in six items to evaluate the risk of bias. Two reviewers rate the studies independently and the final decision is reached by consensus.

Our data shows that the median OS is conspicuously longer in the TTFields group in which the dose of dexamethasone is ≤ 4.1 mg, WMD = 9.23 [95% CI 5.69–12.78]; *P* < 0.05). And the patients whose compliance of TTFields treatment ≥ 75% (≥ 18 h per day) have a significant lower overall survival risk than the patients whose compliance of TTFields treatment < 75% (HR = 0.57 [95% CI 0.46–0.70]; *P* < 0.00001).TTFields is a safe and efficient novel treatment modality. The dose of dexamethasone ≤ 4.1 mg of TTFields treatment and the compliance of TTFields treatment ≥ 75%, ≥ 18 h per day are beneficial to the prognosis of the glioblastoma patients.

## Background

Glioblastoma multiforme (GBM) is the most common type of primary malignant brain tumors and presents a major challenge to the neuro-oncology community [1]. It is characterized by infiltrative growth in adult brain tumors [2] and accounts for approximately 60 to 70% of all malignant gliomas [3]. In the USA, GBM occurs in 3.2 per 100,000 population [4] and the annual incidence increase with age. The treatment of newly diagnosed GBM requires a multidisciplinary approach. Current standard therapy includes maximal safe surgical resection, followed by concurrent radiation with temozolomide (TMZ), and then adjuvant chemotherapy with TMZ [5]. However, with these aggressive treatments, the GBM cannot be completely curable. The average 2-year survival rate is 17.2% and 5-year survival rate is only 5.5% [6]. The widely accepted median survival has been approximately 15 months [7]. Unfortunately, almost all GBM recur after initial therapy with the PFS and OS decreasing apparently, and the majority of patients do not survive beyond 1 year [8]. For patients with recurrent GBM, treatment options are limited, and there is no clear standard of treatment [9]. In order to prolong survival time, treatments become more aggressive including re-surgery, salvage chemotherapy and re-radiation [10]. Hence, there is a critical need for additional treatments for patients with recurrent GBM.

Tumor treating fields (TTFields) has been utilized in many kinds of cancer, such as NCLC, metastatic tumors, and ovarian cancer. TTFields is a unique treatment modality for GBM [11]. The unique mechanism of action of TTFields involves localized delivery of alternating low-intensity, intermediate-frequency, tumor-treating fields via non-invasive transducer arrays attached to the patient’s scalp [12]. TTFields act with a high degree of specificity on rapidly replicating cancer cells, exerting disruptive forces on mitotic spindle formation, resulting in mitotic arrest and cancer cell death. TTFields also exert forces on intracellular organelles and macromolecules during cytokinesis, causing abnormal chromosomal segregation and multinucleation, thus further affecting the replication of daughter cells [11, 13]. Furthermore, these cells also exhibit signs of stress that include elevated cell surface expression of calreticulin, which makes them more readily detectable by phagocytic immune cells, facilitating an immune response against the tumors [14]. In a phase III trial for recurrent glioblastoma (EF-11 trial), TTFields is shown to have equivalent efficacy and less toxicity when compared to Best Physician’s Choice (BPC) chemotherapy [15, 16]. On April 8, 2011, the Food and Drug Administration (FDA) of the United States approved TTFields as a mono therapeutic modality for recurrent GBM based on the results of EF-11 trial [17]. Subsequently, another phase III trial for newly diagnosed GBM (EF-14 trial) demonstrates that the addition of TTFields to maintenance temozolomide chemotherapy vs maintenance temozolomide alone, resulted in statistically significant improvement in progression-free survival (PFS) and overall survival (OS) [18]. As a result, the FDA have approved the use the TTFields for the treatment of newly diagnosed GBM in 2015 [19]. What is more, the National Comprehensive Cancer Network (NCCN) has recommended TTFields with TMZ as a standard Category 1 treatment option for newly diagnosed GBM in 2018 [20]. Except of the promising outcome of TTFields, there are many factors which can affect the efficacy of TTFields, such as KPS, no prior bevacizumab use, dose of dexamethasone, compliance, the extent of surgery and so on [21]. So, we perform a systematic review and meta-analysis of the available evidence to comprehensively determine the impact of compliance and dose of dexamethasone on the efficacy of the TTFields in adult glioblastomas.

### Search strategy

This systematic review and meta-analysis comply with the Preferred Reporting Items for Systematic Reviews (PRISMA) guidelines [22] and the Cochrane Handbook [23]. Three major electronic databases—PubMed, the Cochrane Library, and Embase are searched to identify proper literature reports and trials. We use the following terms in every possible combination: “tumor treating fields” and “TTFields” and “alternative electric fields” and “Novocure” and “NovoTTF-100A” and “glioblastoma” and “GBM” and “malignant glioma” and “compliance” and “dexamethasone” and “Dexasone”. The reference lists of articles identified in initial searches are scanned to obtain additional relevant articles. Two independent reviewers perform the literature search independently. A group discussion with a third investigator is performed to resolve any discrepancies between the two reviewers.

### Study selection and extraction

Inclusion criterion are (1) case reports with ≥ 10, (2) written in English, (3) published from 2000 to 2019, (4) conducted on adult human subjects, (5) reporting outcomes of TTFields on patients with glioblastoma. For each eligible report, we extract the following information: first author’s name, year of publication, country, number of included patients, demographics (mean age, sex), intervention methods, and the endpoints (overall survival OS).

### Quality assessment

The Newcastle-Ottawa Quality Assessment Scale (NOS) [24] is used as an assessment tool to evaluate the methodological quality of all included trials. The scale’s range varies from 0 to 9 stars. Nine stars mean that the included study had the highest quality. Zero stars mean that the included study had the lowest quality. According to the Cochrane Handbook for Systematic Reviews of Interventions [25], articles are graded in six items to evaluate the risk of bias. Two reviewers rate the studies independently and final decision was reached by consensus.

### Statistically analysis

This meta-analysis is done using the RevMan version 5.3 (Nordic Cochrane Centre Cochrane Collaboration, Copenhagen, Denmark). *P* value < 0.05 is considered as significant statistical publication bias. The overall survival (OS) is synthesized using log hazard ratio and its variance to construct point estimates and 95% confidence intervals (CI) [26]. The HR is calculated by log-rank *P*, according to Tierney’s method [27]. Continuous variables are evaluated by means of weighted mean difference (WMD) with its 95% confidence intervals (CI). The standard deviation (SD) is calculated by 95% confidence intervals [28]. The *I*^2^ statistic, which estimates the percentage of total variation across studies attributable to heterogeneity over chance, is used to assess the heterogeneity of the included studies [29]. In the presence of significant heterogeneity (*I*^2^> 50%, *P* < 0.05), a random-effects model is used to calculated data; otherwise, a fixed-effects model was used [30].

### Search hits

**Fig. 1:**
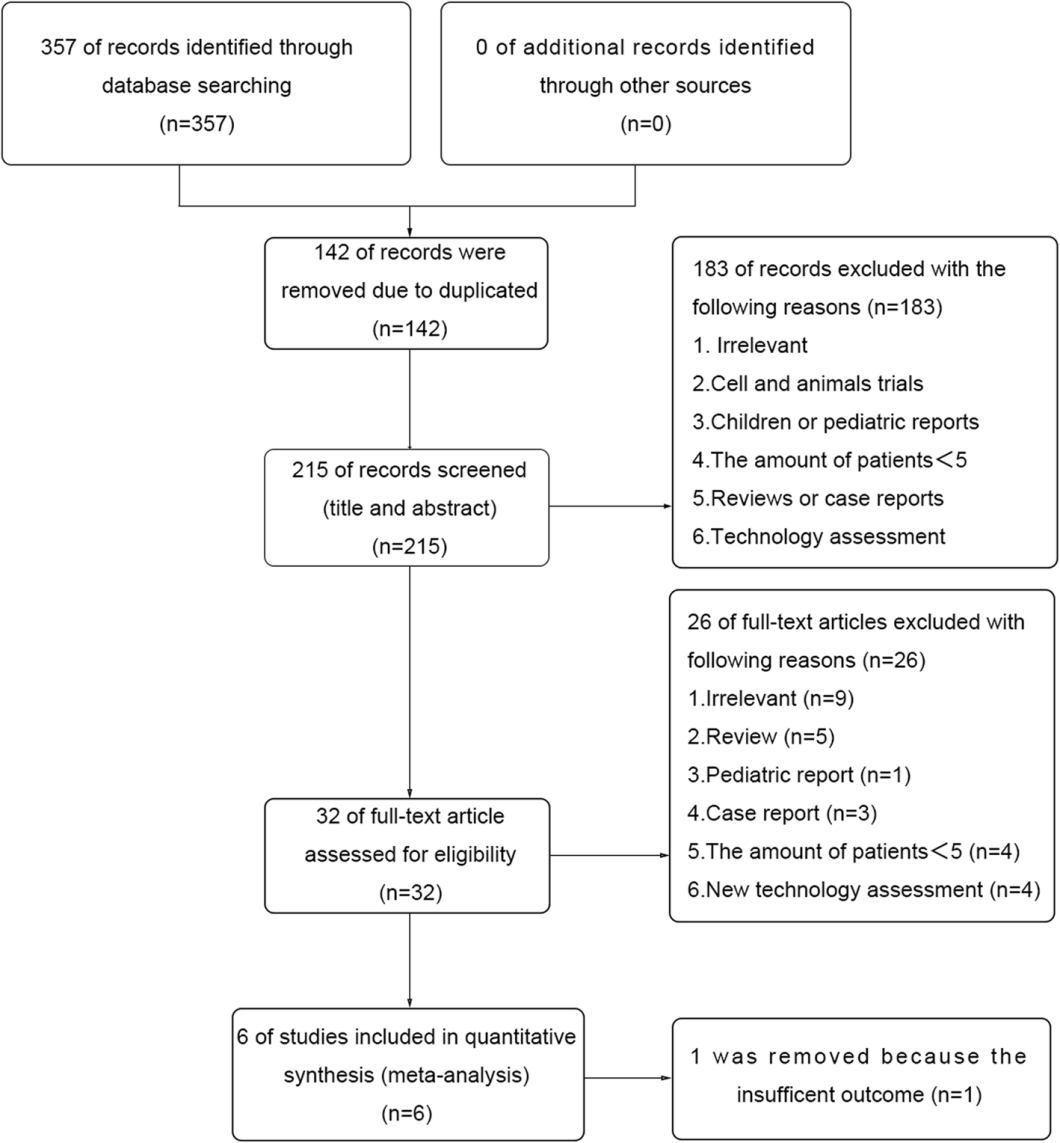
Flow diagram summarizing the selection of eligible articles

The flow diagram of the literature search is shown in Fig. 1 total of 357 studies are identified from PubMed, Embase, and the Cochrane Library. No additional studies are identified from other sources. After removing the duplicated 142 articles, 215 articles are got. According to the exclusion criteria, 183 articles are removed, and leave 32 articles for full-text assessment. After full-text reading, 6 articles remain. Then, 1 of the 6 articles is removed because of the insufficient outcome. At last, 5 articles meet the inclusion criteria and are included in the quantitative analysis. These 5 articles are published between 2014 and 2018.

Three articles are post hoc analysis [14, 16, 31]; one article is randomized phase III trial [18]. One articles are retrospective studies [21]. Three articles are included in the meta-analysis of the compliance of TTFields [18, 21, 31]. The other two articles are included in the meta-analysis of the dose of the dexamethasone on TTFields [14, 16]. All these five articles are showed in Table 1.

**Table 1:** Characteristics of articles included in the meta-analysis

Study ID, year	Journal	Country	Study design	Intervention		Patients, ***n***		Median age	Male, (***n***%)	Median OS (months)	
				T	C	T	C			T	C
Mrugala 2014 [21]	Seminars In Oncology	USA	Retrospective	TTFields with compliance ≥ 75%	TTFields with compliance < 75%	127	160	55 (18~86)	–	13.5	4.0
Stupp 2017 [18]	JAMA	Multinati-onal	RCT	TTFields with compliance ≥ 75%	TTFields with compliance < 75%	265	185	56 (19~83)	316 (68%)	22.6	19.1
Kanner 2014 [31]	Seminars In Oncology	Israel	Post hoc analysis	TTFields with compliance ≥ 75%	TTFields with compliance < 75%	92	28	54 (24~80)	92 (77%)	7.7	4.5
Wong 2014 [16]	Cancer Med	USA	Post hoc analysis	TTFields with daily dexamethasone ≤ 4.1 mg	TTFields with daily dexamethasone > 4.1 mg	14	106	54	–	24.8	6.2
Wong 2015 [14]	British Joutnal Of Cancer	USA	Post hoc analysis	TTFields with daily dexamethasone ≤ 4.1 mg	TTFields with daily dexamethasone > 4.1 mg	56	65	54 (24~80)	92 (77%)	11.0	4.8

### Evidence quality

**Fig. 2:**
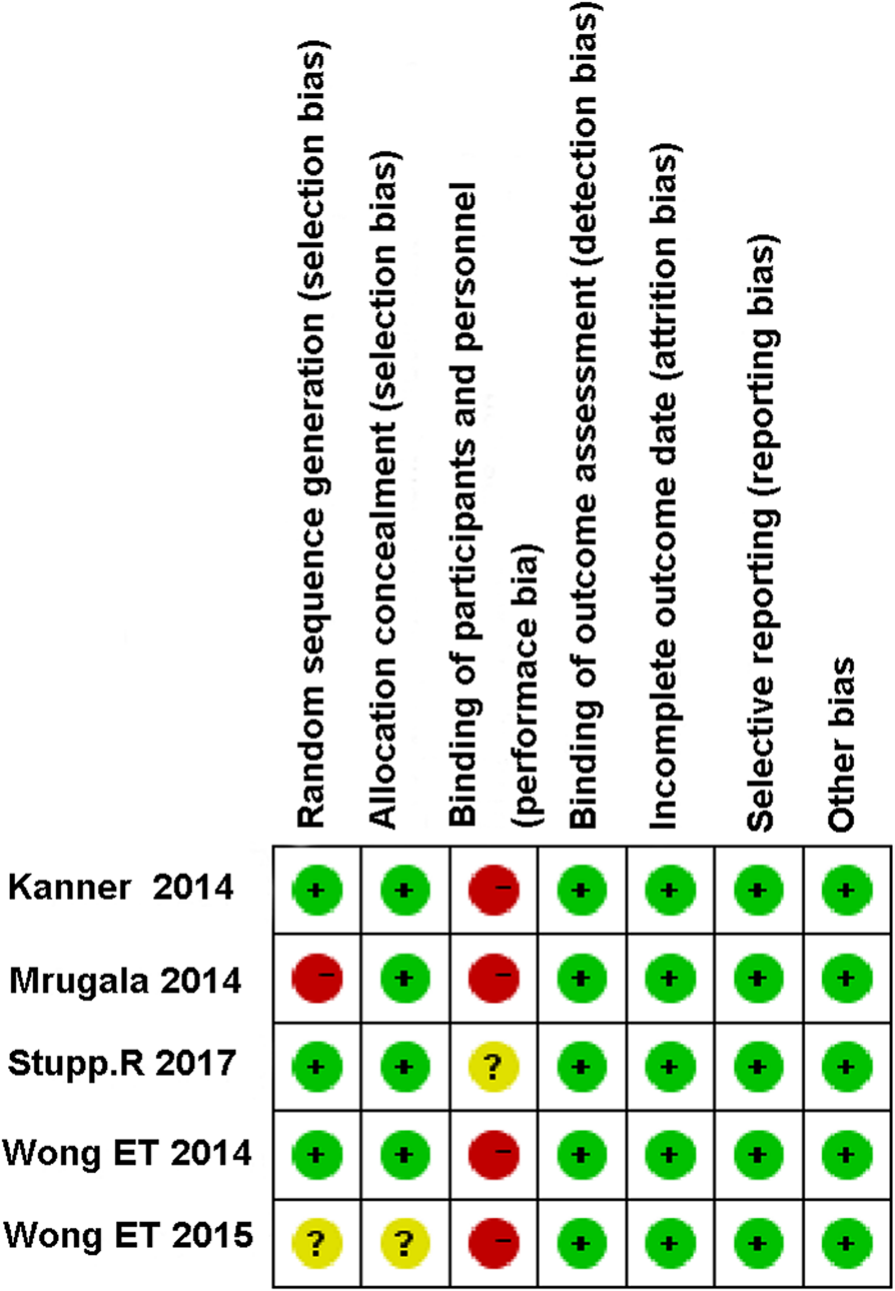
Cochrane Handbook for Systematic Reviews of Interventions

The quality assessment of the five articles, according to the Cochrane Handbook for Systematic Reviews of Interventions, is shown in Fig. 2. Based on the Newcastle-Ottawa Quality Assessment Scale (NOS), Two articles are rated as 6 stars, and three articles are rated as 7 stars. The result is presented in Table 2.

**Table 2:** Newcastle-Ottawa scale for the included articles

Study	Selection	Comparability	Outcome	Score
Mrugala 2014 [21]	***	*	**	6
Stupp 2014 [18]	***	*	***	7
Kanner 2014 [31]	***	*	***	7
Wong 2014 [16]	***	*	**	6
Wong 2015 [14]	***	*	**	7

### Efficacy endpoint

**Fig. 3:**
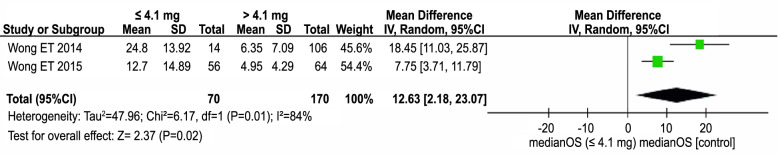
Forest plot of the impact of the dose of the dexamethasone on the efficacy of TTFields

All the five articles involved in the quantitative meta-analysis provided sufficient data for statistical comparisons, two articles are about the dose of the dexamethasone in TTFields treatment, the other three articles are about the compliance of the TTFields treatment. The median OS is conspicuously longer in the TTFields group in which the dose of dexamethasone is ≤ 4.1 mg (WMD 9.23 [95% CI 5.69–12.78]; *P* < 0.05) (Fig. 3). This result reveals that the dose of dexamethasone can significantly impact the efficacy of TTFields. The dose of dexamethasone ≤ 4.1 mg is beneficial to the prognosis of the glioblastoma patients.

**Fig. 4:**
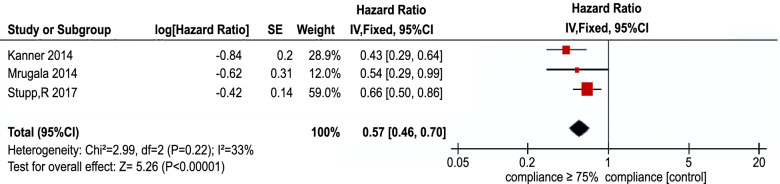
Forest plot of the impact of the compliance on the efficacy of TTFields

Then， analyzing the compliance of the TTFields treatment, the total HR is 0.57 (95% CI 0.46–0.70 *P* < 0.00001) shown in Fig. 4. These results reveal that the patients whose compliance of TTFields treatment ≥ 75% have a significant lower overall survival risk than the patients whose compliance of TTFields treatment < 75%. This proves that the patients whose compliance of TTFields treatment ≥ 75% have an obviously longer OS. The compliance of TTFields treatment ≥ 75% is also beneficial to the prognosis of the glioblastoma patients.

### Publication bias

**Fig. 5:**
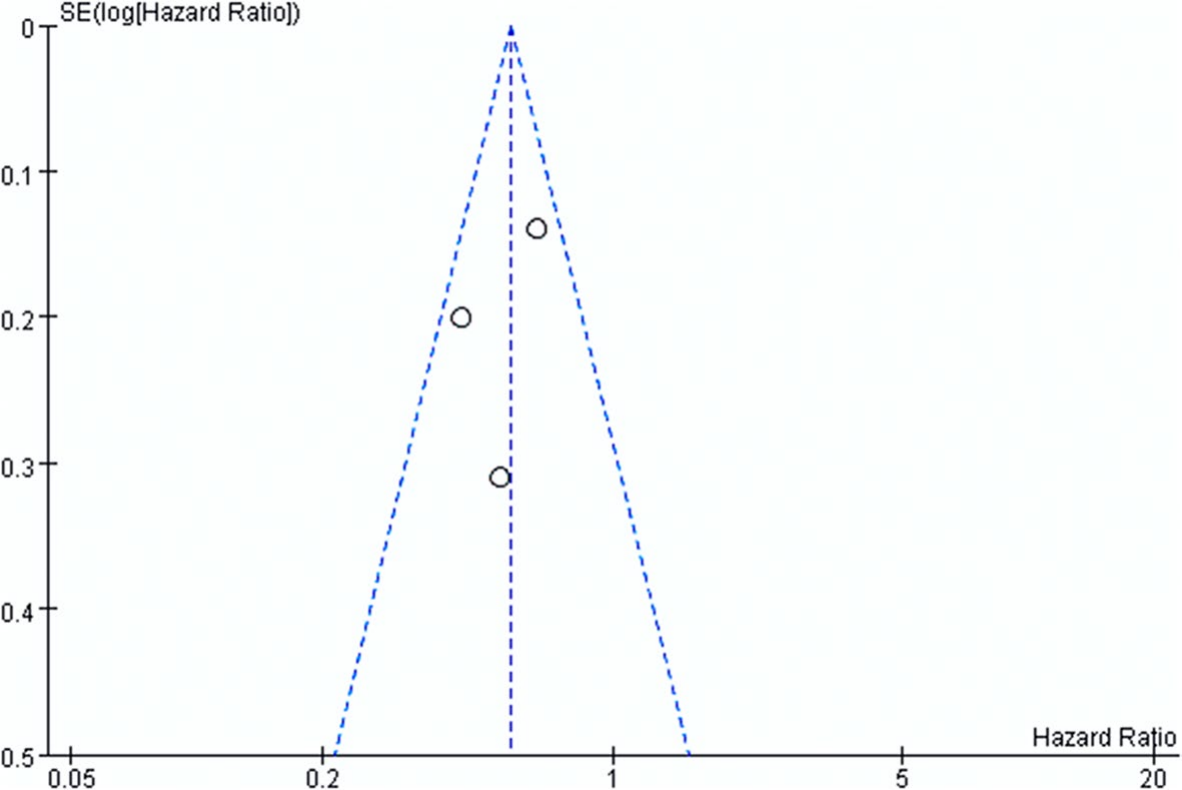
Funnel plot for publication bias test of the compliance of TTFields

Because heterogeneity is high in the analysis of the dose of the dexamethasone (*P* = 0.006 *I*^2^ = 87%), we choose a random effect. The heterogeneity is mainly attributed to the small number of the included studies, thus proposing that more studies are necessary in order to eliminate publication bias. The analysis of the compliance of TTFields treatment has a small heterogeneity as the funnel plot shown in Fig. 5. Egger’s test is not performed due to the small number of the studies that were included [32].

## Discussion

Glioblastoma is a primary malignancy of central nervous system that is very hard to be cured and usually recrudesce. Due to its therapeutic resistance and malignant recurrence, new and innovative therapies are urgently needed for glioblastoma patients. TTFields is a novel cancer remedy which can deliver low-intensity, intermediate frequency (200 kMz) electric fields to the tumor location via some special transducer array [13, 33, 34]. It can disrupt glioblastoma cells during mitosis, leading to apoptosis, aneuploidy, asymmetric chromosome segregation, and make the tumor cells be detected by the immune system more easily. A number of researches have already proven that the use of TTFields is beneficial to the GBM patients [15, 18]. There are numerous factors can affect the efficacy of TTFields [35]. But the relevant researches of the influence factors of TTFields are a little limited. So, this systematic review and meta-analysis identified five articles assessing the dose of dexamethasone and compliance, two important influence factors of TTFields. No similar meta-analysis was identified through literature search.

As Fig. 3 shows above (WMD 9.23 [95% CI 5.69–12.78]; *P* < 0.05), we can find that TTFields treatment with the dose of dexamethasone ≤ 4.1 mg has a longer OS than the group with the dose of dexamethasone > 4.1 mg. As we all know, dexamethasone, a kind of synthetic corticosteroids, is routinely administered to patients with intracranial tumors to alleviate the cerebral edema and provide symptomatic relief [36]. It is also the first-line agents to combat immune-related adverse events [37]. But some researches have revealed that dexamethasone has the potential to cause profound toxicities in patients in large part by suppressing their immune system. In another word, dexamethasone can affect the patient’s antitumor immunity.

Although the immune system has multiple mechanisms to detect and eliminate the tumor cells, tumors can happen when they escape immune surveillance. At this point, the tumors further subvert the immune system by eliciting normal wound healing and tissue remodeling response [38]. In this situation, dexamethasone may enhance existing immunosuppression by global induction of IkBa and inhibition of NF-kB activity in lymphocytes, resulting in global immunosuppression [39]. Then, dexamethasone can lower the number of CD4 lymphocytes in patients with glioblastoma treated with radiation alone or in combination with temozolomide, and this attenuated CD4þ lymphocyte count is associated with increased infections and decreased survival [40]. TTFields treatment can facilitate the immune system to detect the tumor cells. The result of our meta-analysis revealed that dexamethasone exerts an interference on the therapeutic effects of TTFields treatment. The threshold dose at which dexamethasone was able to be used with minimal interact on the TTFields was 4.1 mg per day or lower.

As Fig. 4 shows above (HR 0.57 [95% CI 0.46–0.70] *P* < 0.00001), the patient whose compliance of TTFields treatment ≥ 75% (≥ 18 h daily) have a significant lower overall survival risk than the patients whose compliance of TTFields treatment < 75%. So, our meta-analysis finds that in order to achieve the largest efficacy of TTFields, patients should stay at least 75% of the TTFields therapy time. Ream of experiments have demonstrated TTFields can prolong both the progression-free and overall survival in patients with newly or recurrent glioblastoma. TTFields treatment is a physical modality which is nonchemical, noninvasive treatment and unlike any of the established cancer treatment modalities [41]. TTFields do not have a systemic half-life like oral and intravenous treatment, and it exert the therapeutic effect only on actively dividing cancer cells but not on healthy cells [42]. The therapeutic effect would disappear quickly as soon as the TTFields devices were removed. So, the application of TTFields should be continuous [43]. There are many factors can influence whether a patient decide to accept the TTFields treatment or not. For example, the necessity of hair shaving, frequent array change every 3–4 days, weight of device and spare batteries, visibility of the arrays, increased sweat rate in warm air temperature, alarm tone of the device and problems carrying the device, all the above factors have to be outweighed and might negatively influence compliance of TTFields [44]. As a result, when a patient decides to use the TTFields treatment, the medical staffs should provide the patients with careful education and introduction of this new modality in order to raise the compliance. In our view, an open, fair and honest information provided to the patient is crucial for compliance to therapy [35, 44].

This meta-analysis has some limitations. Firstly, significant heterogeneity could be found in the analysis of dose of dexamethasone, we consider that it is infeasible to eliminate all confounding factors, because of the small amount of the included articles. Secondly, the searching strategy was restricted to articles published in English. Articles with potentially high-quality data that were published in other languages were not included because of difficulties in obtaining accurate medical translation. Thirdly, the amount of the included articles is small, as a result additional high-quality articles are needed for future verifications.

At present, there are still many factors that affect the efficacy of TTFields. Through this study, we found that the dose of dexamethasone and compliance significantly changed the prognosis of patients with glioblastoma. In order to furtherly improve the outcomes of TTFields, more clinical studies and experiments are urgently needed.

## Conclusion

Our meta-analysis identified five studies of TTFields treatment for GBM patient outcomes date. These studies prove that the dose of dexamethasone ≤ 4.1 mg of TTFields treatment and the compliance of TTFields treatment ≥ 75% (≥ 18 h daily) can prolong the glioblastoma patients’ median OS. These results should be interpreted with caution due to the small number of randomized controlled studies. More studies and experiments should be launched in order to explore the newer treatment modality of GBM patients.

## Data Availability

All data generated or analyzed during this study are included in this published article.

## Abbreviations

GBM: Glioblastoma
OS: Overall survival
TTFields: Tumor treating fields
TMZ: temozolomide
BPC: Best Physician’s Choice
FDA: Food and Drug Administration
PFS: Progression-free survival
PRISMA: Preferred Reporting Items for Systematic Reviews
